# Industrial Processing of Algerian Table Olive Cultivars Elaborated as Spanish Style

**DOI:** 10.3389/fmicb.2021.729436

**Published:** 2021-11-04

**Authors:** Chafiaa Sab, Concepción Romero, Manuel Brenes, Alfredo Montaño, Akli Ouelhadj, Eduardo Medina

**Affiliations:** ^1^Laboratory of Food Quality and Food Safety, Faculty of Biological and Agricultural Sciences, Mouloud Mammeri University, Tizi Ouzou, Algeria; ^2^Food Biotechnology Department, Instituto de la Grasa, IG-CSIC, Seville, Spain

**Keywords:** lactic acid fermentation, spoilage, Sevillana, Verdale, Sigoise

## Abstract

Olives from the Sigoise, Verdale, and Sevillana cultivars were elaborated as Spanish-style table olives by four Algerian factories, and the quality and food safety of the industrial table olives have been studied by the analysis of physicochemical and microbiological parameters. Differences were observed between the treatments carried out by the different factories throughout the manufacturing process, especially during the washing stage, but no significant differences were found between the analyzed samples for the concentration of sugars and polyphenols. The final pH values reached at the end of fermentation ranged between 5.04 and 4.27, and the titratable acidity was above 0.4% for all samples. Lactic and acetic acids were produced in mean concentrations of 0.68% and 0.21% as a result of lactic acid bacteria (LAB) and yeast metabolism, respectively. However, the presence of butyric, isobutyric, and propionic acids was also detected, and was related to the growth of undesirable spoilage microorganisms, responsible for secondary fermentations. The high-throughput sequencing of bacterial DNA suggested the dominance of LAB species belonging to genera *Lactiplantibacillus, Leuconostoc*, *Pediococcus*, *Oenococcus*, or *Enterococcus.* The Enterobacteriaceae family was detected during the first days of brining and in only one sample after 120 days of fermentation. Other spoilage microorganisms were found, such as *Lentilactobacillus buchneri* or the *Pectinatus* and *Acetobacter* genera, capable of consuming lactic acid and these played an essential role in the onset of spoilage. The *Clostridium* and *Enterobacter* genera, producers of butyric and propionic acids, were responsible for the malodorous fermentation present in the industrial samples that were analyzed. The study concluded that the safety of the table olives analyzed was compromised by the presence of undesirable microorganisms and microbial stability was not guaranteed. The elaboration process can be improved by reducing the washing steps and the time should be reduced to avoid the loss of fermentable matter, with the goal of reaching a pH < 4.0 after the fermentation and preventing the possibility of the growth of spoilage microorganisms and foodborne pathogens.

## Introduction

Table olives are elaborated from the olive tree’s fruit (*Olea europaea*) and are considered to be the most popular and highly valued fermented vegetable in the Mediterranean countries, having been part of their diet for centuries. Algeria is one of the major table olive-producing countries, with an increased production in recent years estimated at 323,000 tons annually, representing more than 10.5% of the global production (International Olive Council [IOC], 2021).

Raw olives are inedible due to the bitter taste that the polyphenol oleuropein lends them. The main objective of table olives elaboration processes is to remove the bitterness by hydrolysis of the oleuropein, in order to make them palatable (Brenes and de Castro, 1998). There are many different elaboration methods depending on the region, cultivar, the stage of maturity of the olives, etc (Alves and Quintas, 2016; Grounta and Panagou, 2017; Ashaolu and Reale, 2020). Among the different types of commercial table olives, Spanish-style green olives are the most popular elaboration, characterized by an initial alkaline treatment with 2–3% sodium hydroxide (lye) for several hours, which hydrolyzes the oleuropein into non-bitter compounds (Brenes and de Castro, 1998). Then, the fruits are washed twice with tap water to remove the excess alkali, and finally, the olives are covered with brine (9–10% w/v of sodium chloride) to undergo fermentation by autochthonous microbiota for several months (Sánchez-Gómez et al., 2006; Heperkan, 2013).

The increasingly restrictive new environmental policies applied in many countries have forced table olive producers to eliminate the second wash in this type of elaboration, replacing it with a single one, although more intense (12–15 h), to reduce the volume of wastewaters (Sánchez-Gómez et al., 2006). However, Algerian regulations seem to be more flexible in this regard, and factories continue to carry out two washes (Mettouchi et al., 2016). Another trend currently carried out in many factories is to perform a controlled fermentation. Traditionally, fermentation is carried out by autochthonous lactic acid bacteria (LAB) and yeasts but the addition of starter cultures, a practice increasingly employed by processors, reduces the probability of spoilage (Panagou and Tassou, 2006; Rejano-Navarro et al., 2010; Benítez-Cabello et al., 2019; Anagnostopoulos et al., 2020). Also, extensive control of the physicochemical parameters during the table olives elaboration is necessary for an adequate management of the fermentation. The correct sodium chloride concentration, pH values around 4, and free acidity above 0.6% at the end of the process help extend the shelf life, avoid spoilage, and maintain a safe product (Sánchez et al., 2001; International Olive Council [IOC], 2004).

Spanish-style table olives occasionally present with microbial spoilage issues. The uncontrolled growth of certain microorganisms such as Enterobacteriaceae, *Clostridium*, *Propionibacterium* (Medina-Pradas and Arroyo-López, 2015; Grounta and Panagou, 2017), *Cardiobacteriaceae*, *Ruminococcus* (De Castro et al., 2018), and oxidative yeasts (Arroyo-López et al., 2012) can cause secondary fermentations with a consequent increase in the pH value of brines. Spoilage microorganisms associated with table olives can convert the lactic acid, generated by LAB during fermentation, into other organic acids and the formation of off-odors like putrid or rancid butter as a consequence of their metabolisms (Montaño et al., 1992; De Castro et al., 2018). The resulting higher pH values may lead to the proliferation of other foodborne pathogen microorganisms, that were previously inhibited. Improper acidification to pH above 4.6 enables the growth of the spore-forming *Clostridium botulinum*, responsible for the production of the botulinum toxin, generating a risk to the health of consumers (Medina-Pradas and Arroyo-López, 2015). However, table olives have a long history of food safety. The control of the pH, free acidity, and salt concentration in brine would prevent the growth of these non-desirable microorganisms, especially during the summer when temperatures rise (Sánchez-Gómez et al., 2006). Besides, different strategies such as the use of starter cultures to carry out the fermentation under control, the use of preservatives, and heat treatments as pasteurization, together with good manufacturing and hygiene practices, are useful tools as control measures to reduce alterations in table olives (Panagou and Tassou, 2006; Medina-Pradas et al., 2017).

The objective of this work was to study the quality and food safety of the industrial fermented green table olives elaborated as Spanish-style from different Algerian cultivars using the analysis of physicochemical and microbiological parameters.

## Materials and Methods

### Olive Fruits Processing and Sampling

Seventeen samples of brines and olive fruits from different table olive cultivars (Sevillana, Verdale, and Sigoise) elaborated as Spanish-style were collected in February 2020 from four Algerian factories, two of them located in Ain Defla, and the others in Boumerdes and Tizi Ouzou. Samples were collected at different stage of fermentation from industrial fermenters and transported within 24 h to the laboratory for the analysis. The elaboration process was different among the four producers, and the information related to each sample is reported in Table 1.

**TABLE 1:** Characteristic processing of the Spanish-style green olives brines collected from industrial tanks.

Sample	Cultivar	Industry	NaOH concentration (%)	Lye treatment (h)	Washing 1 (h)	Washing 2 (h)	Initial NaCl concentration (%)	Fermentation time (days)
Sig. 1a*	Sigoise	1	1.3	12–14	24	120	16	90
Sig. 1b	Sigoise	1	1.3	12–14	24	120	16	90
Sig. 1c	Sigoise	1	1.3	12–14	24	120	16	60
Sig. 2a	Sigoise	2	2.3	8–10	24	48	12	90
Sig. 2b	Sigoise	2	2.3	8–10	24	48	12	90
Sig. 3a	Sigoise	3	2.5	12	24	48	10	90
Sig. 3b	Sigoise	3	2.5	12	24	48	10	90
Sig. 3c	Sigoise	3	2.5	12	24	48	10	90
Sig. 3d	Sigoise	3	2.5	12	24	48	10	60
Sig. 3e	Sigoise	3	2.5	12	24	48	10	120
Sig. 3f	Sigoise	3	2.5	12	24	48	10	90
Sig. 4	Sigoise	4	2.6	8	24	48	10	120
Ver. 1a	Verdale	1	1.3	12–14	24	120	16	120
Ver. 1b	Verdale	1	1.3	12–14	24	120	16	120
Ver. 2	Verdale	2	2.3	8–10	24	48	12	120
Sev. 1	Sevillana	1	1.3	12–14	24	120	16	120
Sev. 2	Sevillana	2	2.3	8–10	24	48	12	120

Data provided by the industries. *Sample code abbreviation: The first three letters refer to the cultivar, the number refers to the factory, and the last letter refers to the different fermentation vessels analyzed.

In parallel, brine samples of the Sigoise fermentation (Sig. 4) were collected periodically to monitor the fermentation process at 1, 3, 8, 20, 38, 47, and 120 days of fermentation. Also, samples of fruits were collected at the beginning and end of the fermentation process.

### Physicochemical Analysis

Titratable acidity (expressed as % of lactic acid) and pH of brines were measured using a Metrohm 670 Titroprocessor (Herisau, Switzerland). The concentration of NaCl was analyzed by titration with a silver nitrate solution (0.086 N) and expressed as percent (%).

### Analysis of Sugars and Organic and Volatile Acids

Sugars (sucrose, glucose, fructose, and mannitol), organic acids (lactic and acetic acids), and ethanol were extracted and analyzed from olive fruits as described elsewhere (Medina et al., 2007). The concentration of sugars in olive brines was determined by mixing 0.5 ml of brine with 1 g of the acidic resin Amberlite IR-120 and 1 g of the basic resin Amberlite IRA-93, and 1.5 ml of sorbitol (0.05%). After 30 min of occasional agitation, the mixture was filtered through a 0.22-μm pore size nylon filter and injected in the HPLC. The organic acids (lactic and acetic acids) and ethanol were analyzed by filtering 1 ml of brine directly through a 0.22-μm pore size nylon filter before injection. The chromatographic system for the analysis of the sugars and the organic acids was the same as described by Medina et al. (2007). Volatile acid compounds in olive brines (butyric, isobutyric, and propionic acids) were analyzed by HS-SPME-GC-MS following the procedure described elsewhere (Garcia-Villalba et al., 2012). All samples were analyzed in duplicate.

### Analysis of Phenolic Compounds

The phenolic compounds in brines were analyzed as described by Medina et al. (2007). An aliquot of 0.25 ml of brines was diluted in 0.5 ml of distilled water and 0.25 ml of syringic acid (2 mM). The mixtures were filtered through a 0.22-μm pore size nylon filter, and aliquots of 20 μl were injected in the HPLC. All samples were analyzed in duplicate. The results were expressed as the sum of the phenols hydroxytyrosol, hydroxytyrosol 4-glucoside, tyrosol, and salidroside.

### Microbiological Analysis

The viable and culturable microbial population were determined by plating the fermentation brines and their decimal dilutions (in 9 g/L NaCl) with a Spiral Plater (Don Whitley Scientific Ltd., Shipley, United Kingdom). Enterobacteriaceae, mesophilic aerobic bacteria, yeasts, and LAB were counted on VRBG agar (Sigma-Aldrich, St. Louis, MO, United States), Plate Count Agar (Sigma-Aldrich), Yeast Malt Agar with oxytetracycline (Oxoid Ltd., Basingstoke, United Kingdom), and De Man, Rogosa and Sharpe agar (Biokar Diagnostics, Beauvais, France) supplemented with 0.02% sodium azide (Sigma-Aldrich), respectively. Enterobacteriaceae were incubated at 37°C for 24 h, mesophilic aerobic bacteria, yeasts, and LAB were set at 32°C for 48 h, and the numbers of colony-forming units were counted with a Scan 500 colony counter (Interscience, Saint-Nom-la-Bretèche, France).

### Molecular Identification of the Bacterial Population

A selection of four different colonies from VRBG agar plate (sample Ver. 2) were isolated according to their morphology as the main types of bacteria found. The total DNA of the isolates was extracted directly from pelleted cells by the rapid chloroform method described by Ruiz-Barba et al. (2005). The isolates were identified to species level by 16S rRNA gene sequence analysis of a PCR fragment (ca. 1400 bp) amplified using the primer pair 8f (5′-AGAGTTTGATYMTGGCTCAG-3′) and 1492r (5′-GGTTACCTTGTTACGACTT-3′) as previously described in Pérez-Díaz et al. (2019).

### Bacterial DNA Extraction and High-Throughput Sequencing

Culture-independent methods were applied to assess the whole microbiota in selected industrial samples. A volume of 5 ml of brine was spun at 9,000 *g* for 5 min. Then, the pellet was washed twice with saline solution (0.9% NaCl). DNA isolation was performed using the PowerFood^®^ Microbial DNA Isolation Kit (MoBio, Carlsbad, CA, United States) according to the manufacturer’s instructions. Purified DNA samples (∼10 ng/μl) were sent to the Sequencing and Bioinformatic Service of FISABIO (Valencia, Spain) for amplification, library preparation of the 16S rRNA gene of bacteria, and massive sequencing of 16S rDNA amplicons using a MiSeq Illumina platform. Metagenomic libraries, sequencing, and bioinformatic analysis were carried out as described elsewhere (Medina et al., 2018).

### Statistical Analysis

Data were expressed as mean values ± standard deviation. Statistical software version 7.0 was used for data processing (Statistical for Windows, Tulsa, OK, United States). Comparison among mean values was performed by one-way analysis of variance followed by Duncan’s multiple range test, and the differences were considered significant when *p* < 0.05.

## Results and Discussion

The industrial table olive samples were elaborated with a Spanish-style procedure, but the differences between the factories are noticeable, as shown in Table 1. The concentration of the NaOH solution and the duration used for the alkaline treatment ranged from 1.3 to 2.6% and 8–14 h, respectively. It is recommended that the alkaline solution must penetrate 2/3 parts the distance of the pulp to the pit for adequate bitterness removal (Conte et al., 2020). Thus, in those treatments with lower lye concentrations, the duration was longer, and *vice versa*. Differences were reported in the subsequent washing steps, being 24 h for the first washing, and 120 and 48 h for the second washing for factory 1 and 2–4, respectively. The main objective of the washing stage is the removal of the excess of sodium hydroxide from the flesh. Thus, excessive washing causes loss of soluble sugars and polyphenols to be lost from the fruit to wastewaters (Papadaki and Mantzouridou, 2016). Nowadays, the current trend requires the reduction of washing steps to only one with a duration of 6–12 h to minimize the volume of wastewaters generated. Therefore, it seems that the Algerian table olive factories should optimize the duration of both the alkaline and subsequent washing stages.

**FIGURE 1:**
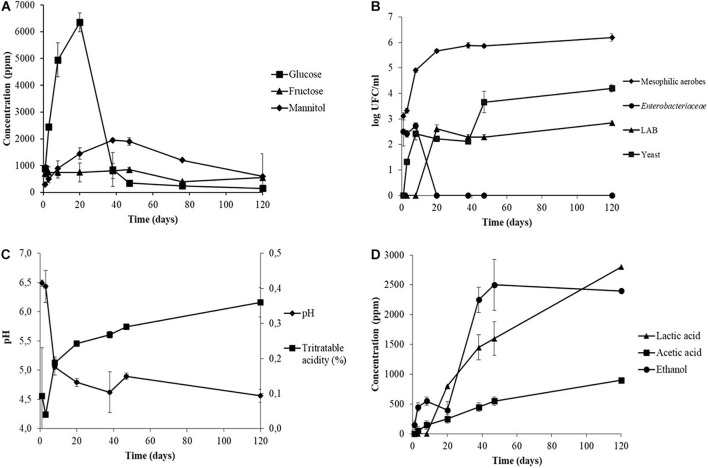
Monitoring of pH and titratable acidity **(A)**, organic acids **(B)**, sugars **(C)**, and viable microbial population **(D)** during the industrial processing of Sigoise cultivar elaborated as Spanish-style green olives (sample Sig. 4). Standard deviation of duplicates is depicted on the error bars.

Glucose was the primary sugar in olive brines, as shown in Figure 1A. The highest concentration was reached at day 20 after brining and it decreased drastically at day 40 when the glucose was entirely consumed until the end of fermentation. The mannitol reached maximum values at around day 40, and its consumption was lower than glucose and decreased at the end of fermentation (Figure 1A). Sucrose was not detected and fructose showed low concentrations at the time of brining (Figure 1A). The sugars of the final fruits showed a reduction of 96.11, 83.88, and 27.83% for the glucose, fructose, and mannitol, respectively, compared to the raw fruit (data not shown). Glucose showed low concentrations or was not detected in the industrial brines listed in Table 2, which implied that it had been consumed during the fermentation (except for the sample Sig. 1a).

**TABLE 2:** Sugars, organic acids, and total polyphenol concentration in industrial Spanish-style green olive brines after fermentation.

Sample	Sucrose (ppm)	Glucose (ppm)	Fructose (ppm)	Mannitol (ppm)	Lactic acid (ppm)	Acetic acid (ppm)	Ethanol (ppm)	Butyric acid (ppm)	Isobutyric acid (ppm)	Propionic acid (ppm)	Total polyphenols (ppm)
Sig. 1a	100 ± 19^a^	4,447 ± 1,398^a^	2,879 ± 171^a^	5,977 ± 467^a^	9,629 ± 27^a^	3,119 ± 42^a^	1,761 ± 66^a^	*N* *D* ^a^	*N* *D* ^a^	14 ± 1^ab^	674 ± 31^a^
Sig. 1b	126 ± 30^ab^	1,905 ± 56^b^	1,126 ± 11^b^	2,848 ± 3^b^	9,551 ± 136^a^	3,125 ± 17^a^	1,709 ± 13^b^	*N* *D* ^a^	*N* *D* ^a^	12 ± 1^ac^	1,071 ± 48^b^
Sig. 1c	119 ± 50^ab^	813 ± 105^cd^	946 ± 116^bc^	3,063 ± 64^b^	8,487 ± 74^b^	2,278 ± 11^bc^	1,554 ± 5^c^	*N* *D* ^a^	*N* *D* ^a^	*N* *D* ^d^	1,447 ± 83^c^
Sig. 2a	115 ± 19^ab^	187 ± 23^cd^	923 ± 42^bc^	3,408 ± 98^c^	5,360 ± 13^c^	2,788 ± 17^d^	4,849 ± 2^d^	*N* *D* ^a^	*N* *D* ^a^	*N* *D* ^d^	2,659 ± 46^de^
Sig. 2b	125 ± 19^ab^	184 ± 13^cd^	810 ± 28^cd^	3,398 ± 47^c^	5,072 ± 142^d^	2,607 ± 143^e^	4,875 ± 21^d^	*N* *D* ^a^	*N* *D* ^a^	*N* *D* ^d^	2,790 ± 39^ef^
Sig. 3a	84 ± 25^a^	599 ± 92^cd^	657 ± 49^ef^	1,373 ± 61^d^	8,721 ± 257^b^	2,153 ± 20^c^	1,347 ± 1^e^	20 ± 2^b^	*N* *D* ^a^	16 ± 5^ab^	1,699 ± 43^g^
Sig. 3b	*N* *D* ^c^	*N* *D* ^d^	490 ± 30^ef^	521 ± 34^efg^	4,159 ± 109^ef^	1,998 ± 4^e^	943 ± 2^f^	56 ± 5^c^	*N* *D* ^a^	6 ± 1^cdef^	1,860 ± 277^h^
Sig. 3c	*N* *D* ^c^	57 ± 13^d^	640 ± 96^def^	346 ± 16^efg^	4,047 ± 134^f^	1,171 ± 7^f^	691 ± 1^g^	49 ± 1^c^	*N* *D* ^a^	21 ± 1^b^	25 ± 27^i^
Sig. 3d	*N* *D* ^c^	*N* *D* ^d^	492 ± 98^ef^	17 ± 1^g^	6,816 ± 15^g^	2,190 ± 11^bc^	619 ± 20^h^	44 ± 0^bc^	*N* *D* ^a^	30 ± 0^g^	2,546 ± 98^di^
Sig. 3e	*N* *D* ^c^	*N* *D* ^d^	542 ± 152^ef^	*N* *D* ^g^	6,683 ± 16^g^	2,148 ± 4^c^	614 ± 7^h^	68 ± 0^c^	*N* *D* ^a^	8 ± 1^acef^	2,361 ± 87^j^
Sig. 3f	148 ± 27^b^	461 ± 20^cd^	942 ± 47^bc^	5560 ± 8^a^	4,380 ± 229^e^	2,176 ± 174^bc^	5,015 ± 10^i^	*N* *D* ^a^	*N* *D* ^a^	2 ± 1^def^	3,374 ± 145^k^
Sig. 4	*N* *D* ^c^	122 ± 43^d^	545 ± 103^ef^	608 ± 59^ef^	2,825 ± 56^h^	864 ± 49^g^	2,414 ± 70^j^	*N* *D* ^a^	*N* *D* ^a^	6 ± 1^cdef^	2,746 ± 98^ef^
Ver. 1a	*N* *D* ^c^	*N* *D* ^d^	385 ± 17^f^	24 ± 34^g^	2,918 ± 169^h^	1,529 ± 18^h^	887 ± 14^k^	*N* *D* ^a^	*N* *D* ^a^	10 ± 0^acf^	2,011 ± 43^l^
Ver. 1b	*N* *D* ^c^	*N* *D* ^d^	451 ± 27^ef^	*N* *D* ^g^	1,914 ± 18^i^	1,704 ± 2^i^	1,621 ± 9^l^	*N* *D* ^a^	*N* *D* ^a^	9 ± 0^acf^	2,695 ± 58^e^
Ver. 2	12 ± 18^c^	*N* *D* ^d^	354 ± 11^f^	63 ± 9^fg^	11,628 ± 18^j^	1,825 ± 14^j^	1,528 ± 5^c^	900 ± 40^d^	3 ± 1^b^	132 ± 13^h^	822 ± 66^m^
Sev. 1	*N* *D* ^c^	937 ± 16^c^	904 ± 159^c^	719 ± 26^e^	14,143 ± 79^k^	2,271 ± 4^bc^	969 ± 7^f^	107 ± 3^a^	1 ± 0^c^	101 ± 1^i^	2,889 ± 72^f^
Sev. 2	*N* *D* ^c^	*N* *D* ^d^	446 ± 130^ef^	27 ± 39^g^	9,138 ± 5^l^	2,297 ± 19^b^	1,147 ± 7^m^	92 ± 0^a^	1 ± 0^c^	125 ± 0^j^	478 ± 12^n^

*Values are the mean of the duplicate analyses ± standard deviation. ND, detected.*

*Column values with the same letter are not significantly different by Duncan’s multiple-range test (*p* < 0.05). Total polyphenols were expressed as the sum of the phenols hydroxytyrosol, hydroxytyrosol 4-glucoside, tyrosol, and salidroside.*

The evolution of the microbial population observed for sample Sig. 4 is shown in Figure 1B. Mesophilic aerobes were the main group detected and reached a population above 5 log CFU/ml in the first days of brining. Instead, the presence of LAB and yeasts were detected after 10–20 days of fermentation and reached values of 2.85 and 4.20 log CFU/ml at the end of fermentation, respectively. Enterobacteriaceae were present during the first days of brining, reaching a population of 4.96 CFU/ml at day 8, and were not detected at day 20 when the pH was below 5 (Figures 1B,C). The population of LAB was very low in comparison to those found by other researchers in the spontaneous table olive fermentation of different cultivars processed as Spanish-style such as Conservolea (Chranioti et al., 2018), Manzanilla and Gordal (De Castro et al., 2018, 2019). Usually, LAB are detected after 48–72 h of brining (Lanza, 2013). Among the factors that could have limited the adaptation of LAB are the brine environment and nutrient availability (Grounta and Panagou, 2017). The low concentration of sugars at the beginning of fermentation and their consumption by other microorganisms like mesophilic aerobes, yeasts, or Enterobacteria delayed the growth of LAB. Conversely, the industrial brines showed a high LAB population in a range of 4.32–7.52 log CFU/ml at the end of fermentation (Table 3), following those observed by previous authors (except for samples Sig. 3f, Sig. 4, and Sev. 1).

**TABLE 3:** Chemical characteristics and viable microbial population of industrial Spanish-style green olive brines after fermentation.

Sample	pH	Titratable acidity (%)	NaCl (%)	LAB (log CFU/ml)	Yeast (log CFU/ml)
Sig. 1a	4.5 ± 0.0^ab^	0.79 ± 0.04^a^	8.4 ± 0.2^a^	6.00 ± 0.29^a^	4.08 ± 0.09^a^
Sig. 1b	4.6 ± 0.0^bc^	0.75 ± 0.04^a^	8.4 ± 0.2^a^	5.44 ± 0.01^b^	4.09 ± 0.16^a^
Sig. 1c	4.8 ± 0.1^cd^	0.66 ± 0.02^ab^	7.4 ± 0.0^a^	4.32 ± 0.22^c^	5.58 ± 0.00^bc^
Sig. 2a	5.0 ± 0.1^e^	0.49 ± 0.09^cb^	6.2 ± 0.4^b^	7.52 ± 0.02^de^	6.24 ± 0.90^cd^
Sig. 2b	5.0 ± 0.0^e^	0.47 ± 0.08^cb^	6.3 ± 0.3^b^	7.57 ± 0.01^de^	5.94 ± 0.23^bcd^
Sig. 3a	4.8 ± 0.1^cd^	0.63 ± 0.05^ab^	4.3 ± 0.1^cde^	7.50 ± 0.01^de^	5.25 ± 0.07^b^
Sig. 3b	4.8 ± 0.1^df^	0.34 ± 0.02^c^	3.2 ± 0.6^cde^	5.74 ± 0.01^ab^	5.95 ± 0.18^bcd^
Sig. 3c	4.4 ± 0.0^ga^	0.43 ± 0.06^cb^	7.6 ± 0.0^a^	6.50 ± 0.28^f^	5.93 ± 0.11^bcd^
Sig. 3d	4.7 ± 0.1^bcd^	0.46 ± 0.07^cb^	3.9 ± 0.1^cde^	6.89 ± 0.71^fg^	6.22 ± 0.79^cd^
Sig. 3e	4.9 ± 0.1^def^	0.27 ± 0.08^c^	4.3 ± 0.2^cde^	5.49 ± 0.27^b^	5.61 ± 0.43^bc^
Sig. 3f	4.5 ± 0.1^ab^	0.61 ± 0.06^ab^	5.8 ± 0.2^b^	2.21 ± 0.01^h^	6.06 ± 0.02^bcd^
Sig. 4	4.6 ± 0.1^b^	0.36 ± 0.04^c^	4.3 ± 0.0^de^	2.85 ± 0.07^i^	4.20 ± 0.15^a^
Ver. 1a	4.3 ± 0.0^bcd^	0.83 ± 0.11^c^	12.6 ± 0.5^cde^	7.25 ± 0.01^dg^	6.32 ± 0.03^cd^
Ver. 1b	4.5 ± 0.0^ef^	0.64 ± 0.07^ab^	5.2 ± 1.6^cde^	7.52 ± 0.02^de^	6.21 ± 0.19^cd^
Ver. 2	4.7 ± 0.1^ab^	0.38 ± 0.05^a^	3.6 ± 0.2^cde^	7.46 ± 0.00^de^	6.20 ± 0.80^cd^
Sev. 1	4.9 ± 0.1^g^	0.63 ± 0.31^a^	4.0 ± 0.1^f^	2.72 ± 0.01^i^	6.66 ± 0.11^d^
Sev. 2	4.6 ± 0.0^ab^	0.74 ± 0.08^ab^	3.8 ± 0.2^be^	7.83 ± 0.01^e^	6.38 ± 0.32^cd^

*Values are the mean of the duplicate analyses ± standard deviation.*

*Column values with the same letter are not significantly different by Duncan’s multiple-range test (*p* < 0.05).*

As expected, sugars were the primary source of carbon for the fermentative microorganisms to produce organic acids during the fermentation process, and consequently, the titratable acidity increased, and the pH decreased (Figure 1C). Most of the industrial samples reached a titratable acidity between 0.43 and 0.85%, but four of them maintained lower values (Table 3). Higher acidity values are usually achieved at the final stage of fermentation, as those found by Lucena-Padrós and Ruiz-Barba (2019) for industrial table olive brines of Manzanilla. Conversely, Chranioti et al. (2018) also found low acidity during the fermentation of Conservolea and it was attributed to the low content of sugars in the brine, as observed in our study. De Castro et al. (2019) noticed acidity values below 0.40% for uninoculated Manzanilla and Gordal table olive brines at 120 days of fermentation, and were corrected with the supplementation of glucose to reach values around 0.60%, extending the fermentation time.

Lactic acid was the main organic acid detected in the industrial table olive brines and was correlated with the evolution of the LAB during the fermentation process due to their fermentative metabolism (Figure 1D). It should be noted that the low concentration of lactic acid generated in the industrial brine samples may be due to the lack of fermentable sugars in the brines (Table 2) caused by excessive washing of the fruits during the elaboration steps (Table 1). Furthermore, the presence of certain polyphenols with antimicrobial activity can inhibit the growth of LAB (Medina et al., 2007). The brine samples showed an average total phenols concentration of 1,821 ppm (Table 2) although the antimicrobial phenolic compounds were not detected. The phenolic content of olives is drastically affected by the alkaline treatment and the successive washing stages during the elaboration as Spanish-style table olives (Medina et al., 2008; Mettouchi et al., 2016; Ait Chabane et al., 2019). In addition, the LAB population showed values below 2.85 log CFU/ml in some specific samples (Table 3), and the high concentration of NaCl of the initial brines could also inhibit the growth of them in favor of yeasts (Arroyo-López et al., 2012). It should be noted that despite the low number of LAB, other groups of microorganisms such as certain Enterobacteriaceae, present in the first step of fermentation, are capable of producing lactic acid although with a lower yield. In studies carried out with Manzanilla, Gordal, and Hojiblanca varieties, researchers observed that the LAB population was more abundant than yeasts, considered to be a good sign of an adequate fermentation (Sánchez et al., 2018; De Castro et al., 2019). The formation of acetic acid and the ethanol in our samples implies a high activity of yeast metabolism. The dominance of yeasts during the fermentation process creates a product with a milder taste and less self-preservation due to a nutrient competition for nutrients with LAB and the subsequent low acid production (Arroyo-López et al., 2012).

The alteration of the aroma and the presence of these bad odors (rancid butter, putrid, etc.) were observed for all the samples at the time of sampling, although they showed different intensities that could not be quantified by a sensorial panel. Among the volatile organic acids, the presence of butyric, isobutyric, and propionic acids in many of the industrial brines (Table 2) must be related to the growth of undesirable spoilage microorganisms (De Castro et al., 2018) whose appearance in table olives is related to an inadequate pH and salt concentration.

Although initial NaCl concentrations were above 10% (data provided by factories), almost half of the samples showed a concentration of NaCl less than 5% (Table 3), which involves a lack of control and correction during the elaboration process. De Castro et al. (2018) noticed that table olive samples with low NaCl concentration developed an off-odor typically deriving from butyric and putrid fermentation. Also, Tataridou and Kotzekidou (2015) considered that NaCl concentration less than 4% induces the formation of a mold layer on the top of the fermentation tank, that also causes alterations in the final table olives. In general, a combination of a salt concentration higher than 5% and a pH below 4.0 are limiting factors of undesirable microbial growth and guarantee correct preservation conditions (Rejano-Navarro et al., 2010; Arroyo-López et al., 2012; Tataridou and Kotzekidou, 2015). As observed in Table 3, the pH values of the samples ranged from 5.0 to 4.3, higher than those reached by other authors for Spanish-style table olives (Tataridou and Kotzekidou, 2015; Lucena-Padrós and Ruiz-Barba, 2019). A slight increase in pH during fermentation could enhance the spoilage produced by Enterobacteriaceae and other microbial groups (Lanza, 2013), and maintaining pH values below 4.6 could also prevent the germination of spores of *C. botulinum* (Valero et al., 2020). Considering the multiple factors that will influence undesirable microbiota, both spoilage and pathogenic, it was deemed relevant to carry out a genomic analysis to know the relative abundance of the different microorganisms present in selected industrial brines samples.

The high throughput sequencing of 16S rRNA gene generated a total of 1,088,785 high-quality sequences with a mean of 136,098 sequences per sample (Supplementary Table 1). Diversity estimators (Shannon, Simpson, and Chao1) were used to analyze the microbial community (Supplementary Table 1). Supplementary Table 2 shows the 63 main bacterial OTUs assigned at family, genus, or species shared among all samples studied.

**FIGURE 2:**
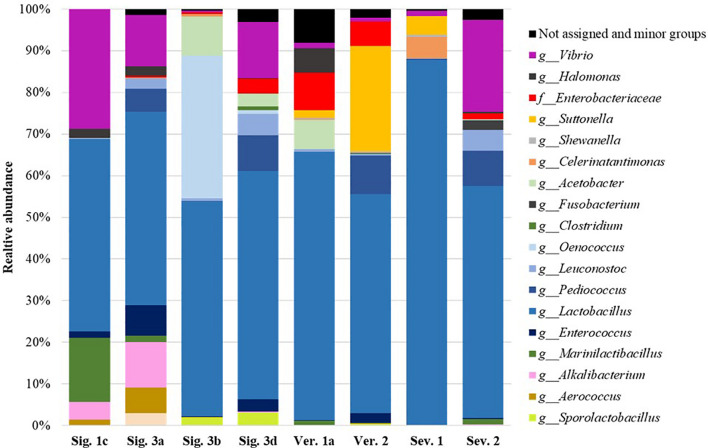
Relative abundance (%) of the bacterial genera or family obtained by sequencing analysis of selected industrial samples after fermentation.

The *Lactiplantibacillus* genus was the predominant bacterial OTU in all the samples analyzed, showing a relative abundance of 87.5–46.2% (Figure 2). This genus has been also identified as the most abundant in natural table olives (Anagnostopoulos et al., 2020; López-García et al., 2021), and in commercial table olives (Benítez-Cabello et al., 2020). The dominance of species such as *Lactiplantibacillus pentosus* and *Lactiplantibacillus plantarum* has been well described in table olive fermentation as the main lactic acid producer of microorganisms, but it is tough to identify at a species level using 16S sequencing (Benítez-Cabello et al., 2019; Bautista-Gallego et al., 2020). Furthermore, other species such as *Levilactobacillus brevis*, *Loigolactobacillus coryniformis*, and *Paucilactobacillus oligofermentans* were detected in the brines with maximum relative abundance values of 8.2, 22.1, and 20.4% for specific samples, respectively. Moreover, other LAB belonging to *Leuconostoc*, *Pediococcus*, *Oenococcus*, or *Enterococcus* genera, previously described by other authors in table olive fermentations (De Castro et al., 2002; Medina et al., 2018; Anagnostopoulos et al., 2020; Benítez-Cabello et al., 2020; López-García et al., 2021), were also detected with a broad representation in the samples.

The Enterobacteriaceae family is frequently found in table olives after brining when pH is still alkaline or neutral and decreases during the first days of fermentation (Lanza, 2013; Anagnostopoulos et al., 2020; Benítez-Cabello et al., 2020). In fact, Enterobacteriaceae were not detected by plating after 20 days in Sig. 4 brine (Figure 1), and in any samples at the end of fermentation, except for the brine Ver. 2 at 120 days, which showed a 3.38 × 10^4^ CFU/ml. However, Enterobacteriaceae were found with high abundance in the samples analyzed by sequencing (Figure 2 and Supplementary Table 2). Benítez-Cabello et al. (2020) also found Enterobacteriaceae present in the most of commercial table olives but with median values below 0.15% of relative abundance. Most of the time, their presence does not cause side effects, but it is well known that certain species or strains can cause illnesses. Selected colonies from VRBD plates isolated from Ver. 2 brine were identified by 16S sequencing as *Providencia rettgeri*, *Providencia vermicola*, and *Proteus vulgaris*, previously identified in Arbequina (Hurtado et al., 2008) and Nocellara Etnea table olive fermentations (Randazzo et al., 2017). These species can cause gastroenteritis and urinary tract infections (Chart, 2012; Wang and Pan, 2014). Apart from the pathogenic species included in this family, these bacteria cause the formation of gas-pocket in the olive fruits and produce metabolites that generate off-odors and off flavors in the final products (Lanza, 2013).

The high pH of some industrial brines above 4.6 (Table 3) denotes that an appropriate lactic fermentation did not occur. Also, linked to the low NaCl concentration, undesirable microorganisms responsible for secondary fermentations could be acting. The presence of *Pectinatus brassicae* (in five out of seven samples analyzed, brines Sig. 3b, Sig. 3d, Ver. 1a, Ver. 2, and Sev. 2) and *Lentilactobacillus buchneri* (in brines Ver. 2 and Sev. 2) (Supplementary Table 2) suggests an initial stage of secondary fermentation as previously described for pickle fermentations (Breidt et al., 2013; Johanningsmeier and McFeeters, 2013; Medina et al., 2016). These microorganisms can metabolize lactic acid, produced by fermentation, into propionic acid, as detected in these brines (Table 2). Likewise, the presence of *Acetobacter* genus in five out of seven brines (brines Sig. 3b, Sig. 3d, Ver. 1a, Ver. 2, and Sev. 2) plays an essential role in the onset of spoilage by converting the lactic acid into acetic acid (Johanningsmeier and McFeeters, 2013; Medina et al., 2016), as well as certain oxidative yeasts (Franco and Pérez-Díaz, 2012; Medina-Pradas and Arroyo-López, 2015). Only the brine sample Sig. 1c did not show butyric, isobutyric, and propionic acids, which is related to the absence of the *Pectinatus* and *Acetobacter* genera (Table 2 and Supplementary Table 2).

Lactic acid consumption in table olives compromises the preservative power of fermented olives and increases the pH values, which can allow for the growth of other undesirable microorganisms detected in the samples as the *Clostridium* and *Enterobacter* genera, also found in some analyzed samples, which can metabolize the lactic acid and generate butyric and propionic acids, respectively (Franco and Pérez-Díaz, 2012; Breidt et al., 2013). Both generate aromas of manure, decomposing organic matter or rancid butter, symptoms of putrid and butyric fermentations (Gililland and Vaughn, 1943) or promote the so-called “zapatería” in spoilt table olives (Kawatomari and Vaughn, 1956; Plastourgos and Vaughn, 1957; Bautista-Gallego et al., 2020). This malodorous fermentation produces olives that are completely unacceptable to the consumer.

From a food safety point of view, most of the brines analyzed in this study showed a pH above 4.6, which could favor the germination of *C. botulinum* spores (Medina-Pradas and Arroyo-López, 2015). Although this species has not been identified in our samples by massive sequencing, these chemical characteristics do not guarantee the safety of the product. Table olives after the fermentation should maintain a pH < 4.0 and the NaCl in brine >5% to prevent any possibility of growth of this foodborne pathogen (Lanza, 2013).

The *Vibrio* genus was also present in all the samples reaching relative abundance even greater than 20% in specific brines (Figure 2 and Supplementary Table 2). It is common to find this genus in table olive fermentation brines and its origin is the salt or marine sources used for the brines (Lucena-Padrós et al., 2015; Medina et al., 2018; Lucena-Padrós and Ruiz-Barba, 2019; Benítez-Cabello et al., 2020). However, within this genus, *Vibrio metschnikovii* has been identified as a pathogenic bacterium associated with human diseases causing gastroenteritis or septicemia (Matté et al., 2007).

In addition to the aforementioned microorganisms, it is also worth highlighting the prominent presence of specific genera in the industrial brines studied, such as the *Celerinatantimonas* genus, previously identified at the end of fermentation in directly brined table olives (Medina et al., 2018; López-García et al., 2021), in commercial table olives (Benítez-Cabello et al., 2020) and as the dominant bacteria in black olive natural fermentation (Penland et al., 2020). The role played by this microorganism during the fermentation of table olives is still unknown, but it was linked to the pH decrease and acetic, citric, and lactic acid production (Penland et al., 2020). Also, the presence of the *Marinilactibacillus* genus, previously identified in green table olive fermentations (Lucena-Padrós and Ruiz-Barba, 2016; López-García et al., 2021), and in commercialized table olive biofilms (Benítez-Cabello et al., 2020), or the *Suttonella* genus, previously found in table olive spoilages, was outstanding (De Castro et al., 2018; López-García et al., 2021).

## Conclusion

The elaboration of table olives is a complex biochemical process in which the indigenous microbiota present in the fruits consume the nutrients to carry out a lactic fermentation to attain proper preservation. In the table olive samples analyzed from the four Algerian factories, adequate pH and acidity values were not reached to guarantee quality and safe table olives. The high pH values and low acidity are consequences of the low initial concentrations of sugars or because its consumption was carried out by other microorganisms different from lactic acid bacteria. High-throughput sequencing revealed the presence of undesirable microorganisms in the cover brines, some of them considered to be foodborne pathogens, so microbial stability is not guaranteed in these fermented table olives.

The Algerian elaboration process can be improved by reducing the washing steps to a single washing (6–12 h), which would avoid the loss of fermentable matter and other biocompounds, and also help to reduce the amount of wastewater originated in the process, thus minimizing the environmental impact. Likewise, the addition of external fermentable matter and/or acid at the beginning of brining is a widespread practice among processors, and would help toward good growth of LAB and inhibit Enterobacteria and other undesirable microbiota, avoiding the secondary fermentations that make the product unacceptable to the consumer. Also, the use of starter cultures favors the growth of LAB, supplanting the undesirable indigenous microbiota and guaranteeing a proper fermentation for the maintenance of the correct preservation conditions. From the point of view of food safety, most of the brines analyzed in this study showed a pH above 4.6, which could favor the germination of *C. botulinum* spores (reference microorganism).

Table olives should maintain a NaCl concentration above 5% throughout the elaboration process, and keep values of pH below 4.0 after the fermentation, the time in which it is recommended that NaCl is raised above 8% to prevent any possibility of growth of spoilage microorganisms and foodborne pathogens, and guarantee high-quality standards.

## Data Availability Statement

The original contributions presented in the study are included in the article/Supplementary Material, further inquiries can be directed to the corresponding author/s.

## Author Contributions

CS, EM, MB, and AO: conception and design of study. CS, EM, CR, and AM: acquisition data. CS, EM, CR, AM, and MB: analysis and/or interpretation of data. CS and EM: drafting the manuscript. CR, MB, AO, and AM: revising the manuscript. All authors contributed to the article and approved the submitted version.

## Conflict of Interest

The authors declare that the research was conducted in the absence of any commercial or financial relationships that could be construed as a potential conflict of interest.

## Publisher’s Note

All claims expressed in this article are solely those of the authors and do not necessarily represent those of their affiliated organizations, or those of the publisher, the editors and the reviewers. Any product that may be evaluated in this article, or claim that may be made by its manufacturer, is not guaranteed or endorsed by the publisher.
