# Supplementary material for: Industrial Processing of Algerian Table Olive Cultivars Elaborated as Spanish Style
Source: Front Microbiol. 2021 Nov 4;12:729436. doi: 10.3389/fmicb.2021.729436 (PMC8600317; doi:10.3389/fmicb.2021.729436)
Supplement: Supplementary file 1 [file Data_Sheet_1.docx]

**Table S1**. Number of reads and genus diversity estimators for 16s rRNA amplicons of olive brine samples.

| **Sample** | **Reads**  **(bp)** | **Mean Length**  **(bp)** | **CHAO1** | **Shannon** | **Simpson** |
| --- | --- | --- | --- | --- | --- |
| Sig.1c | 159494 | 268.16 | 27 | 1.96 | 0.81 |
| Sig.3a | 131349 | 267.43 | 50 | 2.5 | 0.89 |
| Sig.3b | 119920 | 261.08 | 35 | 1.75 | 0.78 |
| Sig.3c | 123497 | 267.43 | 62 | 2.35 | 0.85 |
| Ver.1a | 136713 | 266.49 | 118 | 2.1 | 0.72 |
| Ver.2 | 150079 | 266.37 | 76 | 2.19 | 0.81 |
| Sev.1 | 129046 | 269.69 | 23 | 0.8 | 0.32 |
| Sev.2 | 138687 | 268.72 | 91 | 2.32 | 0.86 |

**Table S2**. Relative abundance (%) of the more representative bacterial OTUs at specie, genus and family level in samples analyzed by 16S sequencing.

| Order | Family | Genus | Species | Sig.1c | Sig.3a | Sig.3b | Sig.3d | Ver.1a | Ver.2 | Sev.1 | Sev.2 |
| --- | --- | --- | --- | --- | --- | --- | --- | --- | --- | --- | --- |
| Bifidobacteriales | *Bifidobacteriaceae* | *Alloscardovia* |  | 0.0 | 0.0 | 0.3 | 1.3 | 0.0 | 0.0 | 0.0 | 0.0 |
| Bacteroidales | *Bacteroidaceae* | *Bacteroides* |  | 0.0 | 0.0 | 0.0 | 0.2 | 0.0 | 0.0 | 0.0 | 0.9 |
|  | *Dysgonomonadaceae* | *Dysgonomonas* |  | 0.0 | 0.0 | 0.0 | 0.0 | 0.2 | 0.0 | 0.0 | 0.1 |
| Flavobacteriales | *Flavobacteriaceae* | *Myroides* |  | 0.0 | 0.0 | 0.0 | 0.0 | 0.2 | 0.1 | 0.0 | 0.1 |
| Campylobacterales | *Arcobacteraceae* | *Arcobacter* |  | 0.0 | 0.0 | 0.0 | 0.0 | 0.0 | 0.2 | 0.0 | 0.1 |
| Bacillales | *Bacillaceae* |  |  | 0.0 | 0.9 | 0.0 | 0.0 | 0.1 | 0.0 | 0.0 | 0.0 |
|  |  | *Halolactibacillus* |  | 0.1 | 3.0 | 0.0 | 0.0 | 0.0 | 0.0 | 0.0 | 0.0 |
|  | *Sporolactobacillaceae* | *Sporolactobacillus* |  | 0.0 | 0.0 | 1.9 | 3.1 | 0.0 | 0.4 | 0.0 | 0.1 |
|  | *Staphylococcaceae* | *Staphylococcus* |  | 0.0 | 0.0 | 0.0 | 0.0 | 0.0 | 0.1 | 0.0 | 0.0 |
| Lactobacillales | *Aerococcaceae* | *Aerococcus* |  | 1.3 | 6.1 | 0.1 | 0.0 | 0.0 | 0.1 | 0.0 | 0.1 |
|  | *Carnobacteriaceae* | *Alkalibacterium* |  | 4.3 | 10.9 | 0.1 | 0.3 | 0.0 | 0.0 | 0.0 | 0.1 |
|  | *Carnobacteriaceae* | *Marinilactibacillus* |  | 15.4 | 1.6 | 0.0 | 0.0 | 1.2 | 0.1 | 0.2 | 1.3 |
|  | *Enterococcaceae* | *Enterococcus* |  | 1.5 | 7.3 | 0.1 | 2.9 | 0.1 | 2.3 | 0.0 | 0.2 |
|  | *Lactobacillaceae* | *Lactobacillus* | *Lactobacillussp.* | 26.2 | 12.6 | 23.3 | 30.9 | 51.0 | 39.6 | 82.2 | 20.1 |
|  |  |  | *Lactobacillusacidipiscis* | 0.3 | 0.0 | 0.0 | 0.0 | 0.0 | 0.1 | 1.5 | 0.1 |
|  |  |  | *Lactobacillusbrevis* | 0.0 | 0.0 | 0.0 | 0.0 | 0.7 | 8.2 | 0.0 | 0.0 |
|  |  |  | *Lactobacillusbuchneri* | 0.0 | 0.0 | 0.0 | 0.0 | 0.0 | 1.0 | 0.0 | 0.1 |
|  |  |  | *Lactobacilluscoryniformis* | 9.5 | 22.1 | 17.1 | 14.8 | 8.2 | 3.3 | 3.4 | 14.1 |
|  |  |  | *Lactobacillusharbinensis* | 0.0 | 0.0 | 0.0 | 0.0 | 0.0 | 0.1 | 0.0 | 0.5 |
|  |  |  | *Lactobacillusmanihotivorans* | 0.0 | 0.0 | 0.7 | 1.0 | 0.0 | 0.3 | 0.0 | 0.3 |
|  |  |  | *Lactobacillusoligofermentans* | 10.2 | 11.9 | 10.4 | 8.0 | 4.6 | 0.0 | 0.3 | 20.4 |
|  |  |  | *Lactobacillusparalimentarius* | 0.0 | 0.0 | 0.0 | 0.0 | 0.1 | 0.0 | 0.0 | 0.0 |
|  |  | *Pediococcus* |  | 0.0 | 0.6 | 0.0 | 2.6 | 0.0 | 2.5 | 0.0 | 1.6 |
|  |  |  | *Pediococcusethanolidurans* | 0.0 | 0.0 | 0.0 | 0.0 | 0.0 | 0.0 | 0.0 | 0.5 |
|  |  |  | *Pediococcusparvulus* | 0.0 | 4.8 | 0.1 | 5.9 | 0.0 | 6.8 | 0.2 | 6.3 |
|  | *Leuconostocaceae* | *Leuconostoc* |  | 0.2 | 2.5 | 0.6 | 5.2 | 0.6 | 0.4 | 0.1 | 5.0 |
|  |  | *Oenococcus* |  | 0.0 | 0.0 | 34.2 | 0.9 | 0.0 | 0.0 | 0.0 | 0.0 |
|  |  | *Weissella* | *Weissellaparamesenteroides* | 0.0 | 0.0 | 0.0 | 0.0 | 0.0 | 0.4 | 0.0 | 0.0 |
| Clostridiales |  |  |  | 0.0 | 0.0 | 0.0 | 0.2 | 0.0 | 0.2 | 0.0 | 0.0 |
|  | *Clostridiaceae 1* | *Clostridium* | *Clostridiumsp.* | 0.0 | 0.0 | 0.0 | 0.0 | 0.0 | 0.0 | 0.0 | 0.0 |
|  |  |  | *Clostridiumpasteurianum* | 0.0 | 0.0 | 0.0 | 0.9 | 0.0 | 0.0 | 0.0 | 0.0 |
|  |  |  | *Clostridiumtyrobutyricum* | 0.0 | 0.0 | 0.0 | 0.0 | 0.0 | 0.2 | 0.0 | 0.0 |
| Selenomonadales | *Veillonellaceae* | *Pectinatus* | *Pectinatusbrassicae* | 0.0 | 0.0 | 0.1 | 0.1 | 0.1 | 0.1 | 0.0 | 0.1 |
| Fusobacteriales | *Fusobacteriaceae* | *Fusobacterium* |  | 0.0 | 0.0 | 0.0 | 0.0 | 0.0 | 0.0 | 0.0 | 2.4 |
| Acetobacterales | *Acetobacteraceae* |  |  | 0.0 | 0.0 | 0.0 | 0.0 | 0.1 | 0.0 | 0.0 | 0.0 |
|  |  | *Acetobacter* |  | 0.0 | 0.0 | 9.4 | 3.0 | 7.1 | 0.1 | 0.0 | 0.1 |
| Rhizobiales | *Rhizobiaceae* |  |  | 0.0 | 0.0 | 0.0 | 0.0 | 0.2 | 0.0 | 0.0 | 0.clostr1 |
| Rhodobacterales | *Rhodobacteraceae* |  |  | 0.0 | 0.0 | 0.0 | 0.3 | 0.3 | 0.2 | 0.0 | 0.4 |
| Sphingomonadales | *Sphingomonadaceae* |  |  | 0.0 | 0.0 | 0.0 | 0.1 | 0.0 | 0.1 | 0.0 | 0.1 |
| Alteromonadales | *Alteromonadaceae* |  |  | 0.0 | 0.0 | 0.0 | 0.0 | 2.2 | 0.0 | 0.0 | 0.0 |
|  | *Celerinatantimonadaceae* | *Celerinatantimonas* | *Celerinatantimonasdiazotrophica* | 0.0 | 0.1 | 0.6 | 0.0 | 0.2 | 0.1 | 5.1 | 0.0 |
|  | *Idiomarinaceae* | *Aliidiomarina* |  | 0.0 | 0.1 | 0.0 | 0.0 | 0.9 | 0.0 | 0.0 | 0.1 |
|  |  | *Idiomarina* |  | 0.0 | 0.0 | 0.0 | 0.0 | 0.3 | 0.0 | 0.0 | 0.0 |
|  | *Pseudoalteromonadaceae* | *Pseudoalteromonas* |  | 0.0 | 0.0 | 0.0 | 0.0 | 0.1 | 0.0 | 0.0 | 0.0 |
|  | *Shewanellaceae* | *Shewanella* |  | 0.1 | 0.2 | 0.0 | 0.0 | 0.2 | 0.3 | 0.6 | 0.1 |
| Cardiobacteriales | *Cardiobacteriaceae* | *Suttonella* |  | 0.0 | 0.0 | 0.0 | 0.0 | 0.0 | 0.3 | 0.0 | 0.0 |
|  |  | *Suttonella* | *Suttonellaornithocola* | 0.0 | 0.0 | 0.0 | 0.0 | 1.9 | 24.9 | 4.5 | 0.0 |
| Cellvibrionales | *Cellvibrionaceae* | *Marinimicrobium* |  | 0.0 | 0.0 | 0.0 | 0.0 | 0.2 | 0.0 | 0.0 | 0.0 |
| Enterobacteriales | *Enterobacteriaceae* |  |  | 0.0 | 0.4 | 0.5 | 3.4 | 6.6 | 2.0 | 0.0 | 0.9 |
|  |  | *Citrobacter* |  | 0.0 | 0.0 | 0.0 | 0.0 | 0.0 | 0.1 | 0.0 | 0.4 |
|  |  | *Enterobacter* |  | 0.0 | 0.0 | 0.0 | 0.1 | 1.5 | 0.9 | 0.0 | 0.1 |
|  |  | *Mangrovibacter* |  | 0.0 | 0.0 | 0.0 | 0.1 | 0.9 | 0.0 | 0.0 | 0.0 |
|  |  | *Serratia* |  | 0.0 | 0.0 | 0.0 | 0.0 | 0.0 | 2.9 | 0.0 | 0.0 |
| Nitrosococcales | *Methylophagaceae* | *Methylophaga* |  | 0.0 | 0.0 | 0.0 | 0.0 | 0.6 | 0.0 | 0.0 | 0.0 |
| Oceanospirillales | *Halomonadaceae* |  |  | 0.0 | 0.0 | 0.0 | 0.0 | 0.3 | 0.0 | 0.0 | 0.0 |
|  |  | *Halomonas* |  | 2.2 | 2.3 | 0.0 | 0.2 | 5.9 | 0.0 | 0.0 | 0.4 |
|  |  | *Salinicola* |  | 0.0 | 0.1 | 0.0 | 0.0 | 0.6 | 0.1 | 0.0 | 0.3 |
|  | *Nitrincolaceae* | *Marinobacterium* |  | 0.0 | 0.0 | 0.0 | 0.0 | 0.0 | 0.0 | 0.4 | 0.0 |
| Pseudomonadales | *Moraxellaceae* | *Acinetobacter* |  | 0.0 | 0.0 | 0.0 | 0.0 | 0.0 | 0.0 | 0.0 | 0.0 |
|  | *Pseudomonadaceae* | *Pseudomonas* |  | 0.0 | 0.0 | 0.0 | 0.5 | 0.0 | 0.0 | 0.0 | 0.0 |
| Vibrionales | *Vibrionaceae* | *Vibrio* | *Vibrio sp.* | 28.3 | 11.1 | 0.3 | 13.2 | 1.3 | 1.0 | 1.2 | 17.1 |
|  |  |  | *Vibrio metschnikovii* | 0.4 | 0.4 | 0.0 | 0.3 | 0.0 | 0.0 | 0.0 | 5.0 |
|  |  |  | *Vibrio penaeicida* | 0.0 | 0.8 | 0.0 | 0.0 | 0.0 | 0.0 | 0.0 | 0.0 |
| Not assigned and minor groups | | | | 0.0 | 0.1 | 0.0 | 0.4 | 1.6 | 0.5 | 0.0 | 0.4 |
